# Multicolor Luminescence of a Polyurethane Derivative Driven by Heat/Light-Induced Aggregation

**DOI:** 10.1021/acs.macromol.3c01345

**Published:** 2023-09-29

**Authors:** Nan Jiang, Ke-Xin Li, Wei Xie, Shu-Ran Zhang, Xin Li, Yue Hu, Yan-Hong Xu, Xing-Man Liu, Martin R. Bryce

**Affiliations:** † Key Laboratory of Preparation and Applications of Environmental Friendly Materials, Key Laboratory of Functional Materials Physics and Chemistry of the Ministry of Education, Jilin Normal University, Changchun 130103, China; ‡ School of Chemistry and Chemical Engineering, Ningxia University, Yinchuan 750021, China; § Department of Chemistry, Durham University, Durham DH1 3LE, U.K.

## Abstract

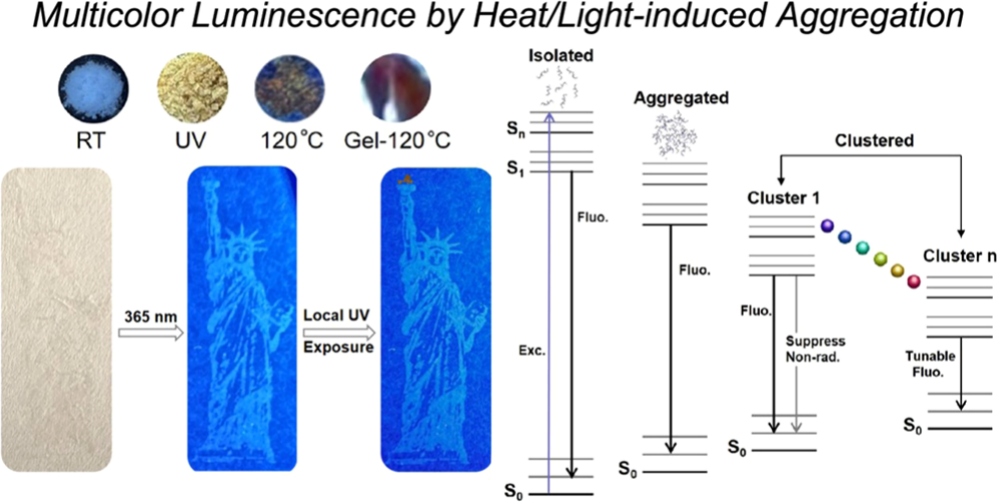

The study of aggregate formation and its controllable effect on luminescence behavior has a far-reaching influence in establishing a universal aggregation photophysical mechanism. In this paper, we obtained clusters with different extents of aggregation by heat-induced or light-triggered aggregation of a new polyurethane derivative (**PUE**). The controllable regulation of multicolor fluorescence of a single (nondoped) polymeric material is realized. The luminescence behavior of **PUE** varies with microscopic control of the aggregation structure. Compared with the powder state, the enhanced atom–atom and group–group interactions of **PUE-gel** effectively limit the nonradiative transitions in the excited state and result in a red-shift in emission. This work avoids complex organic synthesis and demonstrates a simple strategy to induce aggregation and regulate the emitting color of macromolecules, providing a template for developing new materials for multicolor fluorescence. In addition, a pattern was constructed with encryption, anticounterfeiting, and information transmission functions which provide a proof-of-concept demonstration of the practical potential of **PUE** as a smart material.

## 1 Introduction

Ever since the 1660s when Newton advanced the understanding of the color of light and ushered in a new era of scientific endeavor, humans have continually pursued new light-induced phenomena and applications. Compared with small organic molecules that often require complex chemical synthesis and tedious purification, macromolecular luminescent materials have a wide range of applications in the fields of display technologies, photochemical sensing, information storage, and photoelectric devices because of their advantages such as easy functionalization, low-cost high-volume production, and diverse molecular conformations.^1−5^ The luminescence of molecules is not only related to their intrinsic molecular structure but also to their aggregation state.^6−10^ Thus, the in-depth study of aggregate luminescence is of great significance to guide further developments in functional macromolecular materials. Polyurethanes are a class of block polymers formed by alternating soft and rigid segments incorporating urethane (carbamate) links.^11−13^ Such a structure not only provides a large number of lone pair electrons but is also rich in hydrogen bonding and other noncovalent interaction sites.^14−18^ Polyurethane is a suitable material template for in-depth studies of complex spatial interactions and aggregation’s luminescence behavior.

In this work, we designed and synthesized a polyurethane derivative. By exploiting heat/light-induced aggregation, microscopic control of the structure and remote control of the macroscopic luminescence behavior of the material have been realized. Thermochromic/photochromic functional materials exhibit dynamic multicolor fluorescence changes under heat/light stimulation.^19−23^ Compared with other stimulus-response materials, they have the advantage of contact-less control,^24−26^ which endows them with great potential applications in the fields of optical response materials,^27−29^ hologram photography,^30−32^ encryption, and anticounterfeiting.^33−35^ There have been some studies on thermo-photoluminescent oligo/polyurethanes, but most of them are composites, such as doping with metal ions, SiO_2_, or rare-earth luminous complexes.^36−40^ There are also several pure polyurethanes with thermal/photochromic properties obtained by covalent linking or simply doping with monomers that can undergo isomerization.^41−45^ However, such systems are often plagued by aggregation-caused quenching (ACQ) effects. For example, after gelation, the dense three-dimensional network would impose significant steric hindrance, which makes it challenging to isomerize the chromophores.^46^ As a result, they cannot exhibit thermal or photochromic behavior in the aggregate state, which greatly limits the application of such materials.

Herein, we report a new nonconjugated polyurethane derivative (**PUE**) based on estradiol. Estradiol was chosen as a readily available backbone unit to provide some defined aliphatic structural rigidity in the polymer. **PUE** shows thermal and photochromic responses through simple aggregation changes. Combining the advantages of gelation- and aggregation-induced emission (AIE), **PUE**’s multicolor fluorescence range broadens from blue to red. As an AIE system without typical π-conjugated units, a systematic study of luminescence properties of **PUE** in solution, powder, and gel was undertaken to probe the relationship between noncovalent interactions in different aggregation states and the macroscopic luminescence properties of the material. The development of AIE soft materials of this type with multicolor luminescence will facilitate the construction of new advanced multifunctional optical materials.

## 2 Results and Discussion

### 2.1 Synthesis

**Figure 1:**
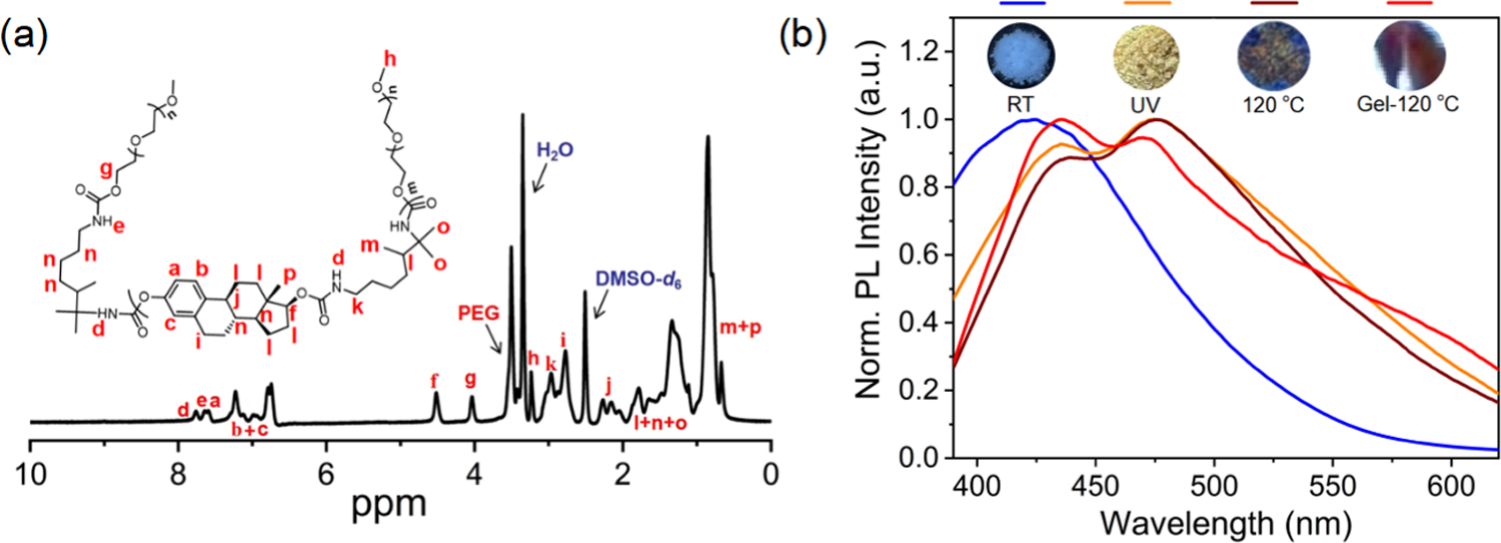
(a) Molecular structure and ^1^H NMR spectrum of **PUE** in DMSO-*d*_6_, with residual proton resonance in DMSO-*d*_6_ (δ = 2.5 ppm). (b) PL spectra of **PUE-powder** at room temperature; after ultraviolet radiation; and heated at 120 °C and **PUE**-**gel** heated at 120 °C (λ_ex_ = 365 nm). Inset: images of these samples under a 365 nm UV lamp.

The synthesis and characterization of **PUE** are described in Section 4 and in the Supporting Information (SI). Figure 1a shows the molecular structure and ^1^H NMR spectrum of **PUE**.

### 2.2 Physical Properties

**Figure 2:**
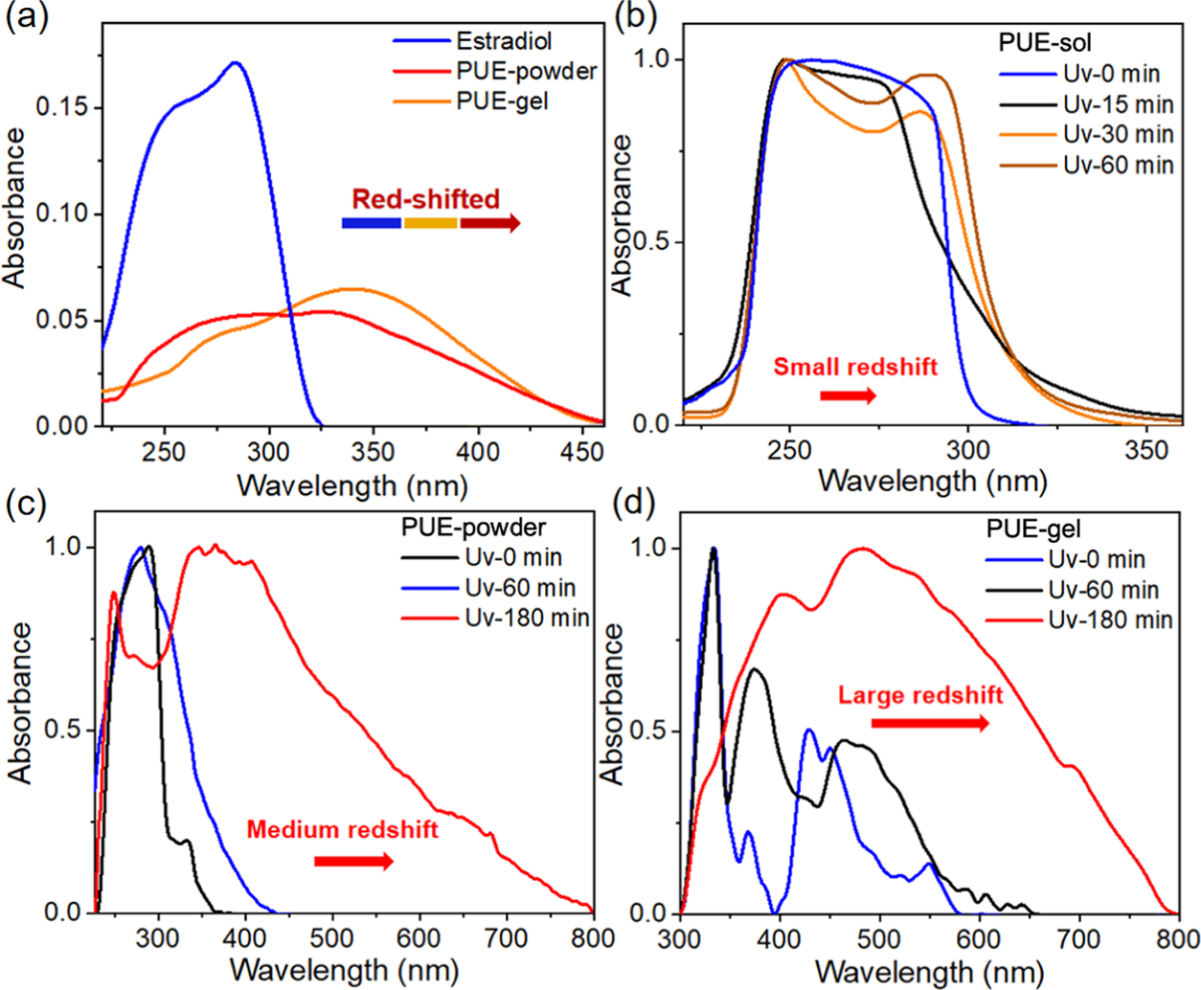
(a) UV–vis spectra of estradiol monomer, **PUE-powder**, and **PUE-gel**. (b) UV–vis spectra of **PUE-sol** before and after ultraviolet radiation. (c) UV–vis spectra of **PUE-powder** before and after ultraviolet radiation. (d) UV–vis spectra of **PUE-gel** before and after ultraviolet radiation.

As shown in Figure S2 in the SI, based on the emission spectra, the estradiol monomer could not be excited as a chromophore to emit visible light. However, after polymerization, the nonconjugated product (**PUE**) based on estradiol displayed typical cluster aggregation-induced emission characteristics. As shown in Figure S3a in the SI, as the excitation wavelength increased from 320 to 540 nm, the fluorescence emission of the **PUE-powder** gradually red-shifted. Figure S3b shows that the emission intensity of **PUE-sol** in the trichloromethane solution increased with increasing concentration within the range of 1.56 × 10^–5^ to 5 × 10^–4^ M. These results indicate that **PUE** is a cluster luminescent material with excitation dependence and concentration dependence, features typical of AIE materials.^47^Figure 2a shows that the estradiol monomer does not show absorption in the visible region. However, after the polymerization reaction, the absorption of the material was greatly shifted into the visible region. After gelation, due to the unique cross-linked network structure, the absorption maximum of the **PUE-gel** was further red-shifted.

Surprisingly, **PUE** exhibits multicolor fluorescence driven by heat or ultraviolet (UV) light over a large color gamut span (Figure 1b), which is consistent with the absorption spectra (Figures 2 and S4). Figure 2b–d shows that the absorption of **PUE-sol**, **PUE-powder**, and **PUE-gel** varies greatly after UV irradiation, indicating that **PUE** has obvious photochromic properties. Compared with **PUE-sol**, the absorption range of **PUE-powder** and **PUE-gel** was much larger, implying that aggregation can amplify the photochromic effect. For solution, powder, and gel samples, different heating/illumination temperatures and times are required to achieve aggregation. Weakly aggregated solution samples of **PUE-sol** need only 1 h of illumination to change the fluorescence from blue to yellow (Figure S9a). For the powder, the fluorescence change can be observed by heating for 3 h, but for the gel sample with strong initial aggregation, a longer time (5 h) is required to achieve the fluorescence transition. It is worth noting that heating samples at 80 °C for a few hours and then placing them into a 120 °C oven will achieve a fluorescence transition faster than simply heating at 120 °C, implying that the aggregation behavior is gradual and requires time for the physical change to occur.

### 2.3 Mechanism of Multicolor Luminescence

**Figure 3:**
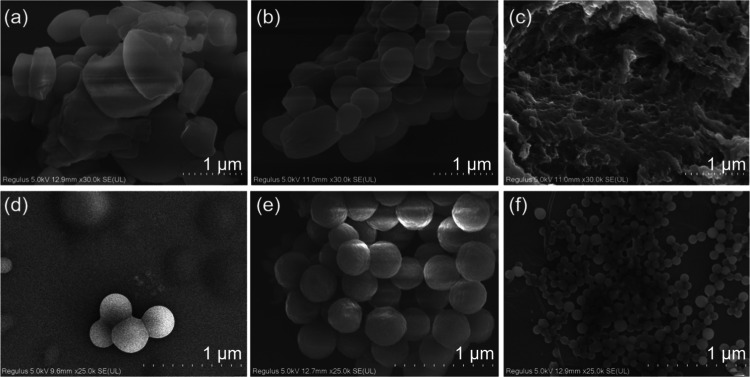
SEM images of **PUE-powder** dispersed in ethanol (a) at room temperature (b) heated at 85 °C and (c) heated at 120 °C. SEM images of **PUE-gel** dispersed in ethanol (d) at room temperature (e) heated at 85 °C and (f) heated at 120 °C.

In order to explore the mechanism of the thermochromism, the ^1^H NMR spectra (Figure S5a) showed that the chemical structure of **PUE** did not change after high-temperature treatment and ultraviolet irradiation, which ruled out the possibility that the fluorescence changes are due to isomerization or degradation of the molecular structure. However, heating has an impact on the aggregation behavior at the supramolecular level of physical change (Figures S6 and 3). We propose, therefore, that the unusual thermal/photochromic properties of **PUE** arise from changes in the molecular aggregation state of **PUE**: the discrete molecular chains of **PUE** do not emit at long wavelength, but the aggregates of **PUE** in the solid powder and gel state can achieve a higher degree of spatial electron conjugation and finally obtain the long-wavelength emission, which is a feature of nonconjugated cluster aggregation luminous materials. To probe this mechanism, the material morphology was studied by scanning electron microscopy (SEM). **PUE-powder** at room temperature and after heating was dispersed in ethanol. SEM results (Figure 3a–c) showed that compared to room temperature, the sample heated at 85 °C showed a denser nanosphere microstructure at the same magnification. In addition, **PUE-powder** treated at 120 °C showed a more concentrated gelated cross-linked structure. This is consistent with the thermochromic behavior of **PUE-powder**: the more it aggregates, the more red-shifted the emission. Figure S6c,d shows that UV irradiation can also induce nanoaggregation. The excitation energy generated by UV light can induce the formation of aggregates,^46^ and in addition, significant photothermal interactions can be produced by UV light irradiation, which can also promote nanoaggregation.^48^

Due to the appearance of nanoaggregates, the energy level of **PUE** is split, gradually reducing the gap, and, concomitantly, the luminescence is gradually red-shifted. We suggest that the dispersion medium in the gel network provides room for the clusters to move and helps regulate their luminous properties by manipulating the intramolecular motion. Therefore, **PUE-gel** has more obvious thermochromic and photochromic properties than **PUE-powder**. Figure 3d–f shows the SEM studies of the aggregation behavior of the gel. At room temperature, **PUE-gel** presents a dispersed nanosphere structure. However, after heating at 85 °C, a very tightly clustered “bunch of grapes”-like nanosphere structure is observed (Figure 3e), and the uneven surface of the nanospheres could be clearly observed at higher magnification (Figure S6a). After heating to 120 °C, the nanospheres split into smaller nanospheres and even formed single-ended aggregated nanorods (Figure S6b). The above experimental results prove that thermogenic and photochromic processes occur through nanoaggregation of **PUE**. A large number of electronic interactions between chromophores enhance the overlap of excited-state orbitals, leading to energy-level splitting. Clusters with different extents of aggregation bring different energy gaps, ultimately leading to the multicolor emission behavior of **PUE**.

We propose that the aggregation behavior of **PUE** is due to its conformational changes. There are many noncovalent interaction sites on the **PUE** chain, which are critical for aggregation-induced polychromatic fluorescence. The crystallization rate of **PUE-powder** before and after heating was studied by wide-angle X-ray diffraction (WAXD). As shown in Figure S5b, the broad peak at 2θ around 16.8° corresponds to the PU skeleton, as reported previously, indicating that both crystalline and amorphous structures exist in **PUE** samples.^18^ The diffraction peak intensity of **PUE-powder** increased and widened with increasing temperature, indicating that the chains’ conjugation length increased with a transition to a more conformational order. At room temperature, due to the flexible chain structure and extensive intramolecular hydrogen bonding, the chains are relatively twisted and coiled. However, upon heating, a substantial number of intrachain heat-sensitive hydrogen bonds break, and the **PUE** chains partly extend. This favors the increase of interchain interactions, which dominate the strong aggregation behavior. Thus, a large number of disordered amorphous chains are transformed into more ordered crystalline conformations with increasing conjugation length, red-shifting the emission, in agreement with the PL results discussed above (Figure 1b).

Analysis of the Fourier transform infrared (FT-IR) spectra provides strong support for this conjecture. As shown in Figure S7, the FT-IR spectra of **PUE-powder** at 0 and 120 °C showed that the N–H stretching peaks were significantly broader and shifted from ν_max_ 3341 to 3429 cm^–1^ after heating. At the same time, the C=O bond moved from ν_max_ of 1718 to 1628 cm^–1^. **PUE-powder**, **PUE-gel**, and **PUE-sol** after UV irradiation showed changes similar to those of **PUE-powder** after heating (Figure S8). These wavenumber changes show that the hydrogen bond between the N–H and C=O units weakened after exposure to heat and UV light. This can be explained by a loose aggregated structure with predominant intramolecular H-bonds at room temperature. After heating or UV irradiation, these bonds are weakened or destroyed, and noncovalent interactions between adjacent chains become dominant, thereby enhancing the aggregation of **PUE** chains through noncovalent spatial conjugation, which causes the luminescence to red-shift further. A detailed discussion is in Section 4.4.

Furthermore, the FT-IR and emission spectra of the photochromic **PUE** solution have been studied after storage in daylight at room temperature for 1 day (Figures S8d and S9a). It was found that **PUE-sol** returned to the blue fluorescence state it had before exposure to UV light and the intramolecular hydrogen bonding had also been restored. **PUE-sol** has a faster color-changing/color-reverting process than **PUE-gel** (Figures S8c and S9b); the latter has increased stability after photochromism because of the tight three-dimensional cross-linked network structure of the gel (Figure S8).

In order to better understand the cluster aggregation luminescence properties of **PUE**, the luminescence lifetime and quantum efficiency (QY) of **PUE-sol**, **PUE-powder**, and **PUE-gel** were recorded (Table S1). From the analysis of the experimental results, it can be seen that UV light can greatly increase the fluorescence lifetime of **PUE-sol**, which may be due to the formation of various clusters, resulting in energy-level splitting and the increase of radiative transition channels. For powdered samples, the QY and lifetimes are significantly reduced after heating, which may be because of the formation of multitype clusters in the solid aggregation state; although the energy level is split, this also enhances the radiative transition between the n_th_ singlet electron excitation states (S*_n_*) to the ground (S_0_) state and weakens the rigidity of the material, finally leading to the decrease of lifetime and QY. For the gel sample, the existence of solvent molecules in the initial state obstructs the electronic communication between the luminous clusters, so the lifetime and efficiency are low. However, after heating at 80 °C and the evaporation of solvent molecules, the efficiency of **PUE-gel** reaches 8%, which is because compared with the powder sample, the efficiency of **PUE-gel** reaches 8%. This is because the initial **PUE-gel** molecule has a tighter structure, and when the solvent evaporates, the space electrons are fully conjugated, resulting in a significant increase in efficiency. However, further heating will cause the sample to undergo a similar process to the powder sample, so the efficiency becomes lower again. We speculate that there may be different rules for the aggregation behavior of cluster luminescent materials for different initial states of samples, which may lead to unexpected results, such as a large increase in luminous efficiency. Further exploration of this aspect is beyond the scope of the present manuscript, but it can be expected that more detailed explanations will be established in the future. These results provide an effective bridge for further understanding the relationship between molecular aggregation and macro-luminescence properties.

### 2.4 Theoretical Calculations

**Figure 4:**
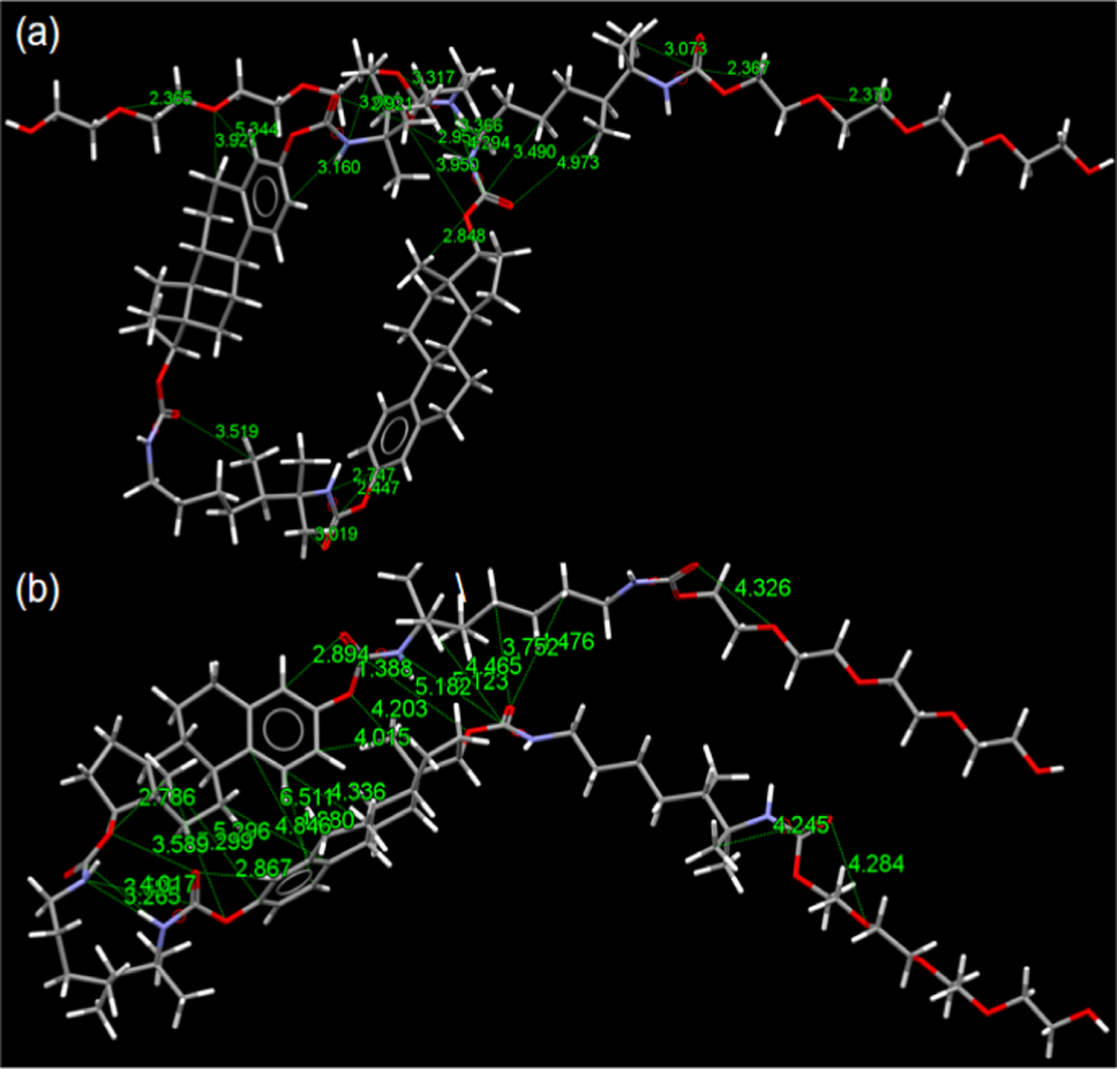
Theoretical calculations based on the single **PUE** chain by the B3LYP/6-31g(d) method in different degree folding modes. (a) Helical conformation. (b) Folded conformation.

In order to gain insight into the origin of the aggregation-induced polychromatic fluorescence of **PUE**, the photophysical mechanisms involved were further explored at the molecular level. Density functional theory (DFT) was used to optimize the **PUE** chains under different folding modes. The corresponding through-space interaction (TSI) diagrams are visualized in Figures 4 and S10–S12. Due to the softness of the polymer chains, there were strong intramolecular TSIs in a single **PUE** chain (e.g., C–H···π; C–H···O=C; π···π; C–H···N; N–H···N). Clearly, aggregation behavior mainly depends on intermolecular rather than intramolecular interactions.^4^ Thus, these strong intrachain TSIs result in loose aggregation structures, and the original **PUE** shows blue fluorescence. However, after heating or UV irradiation, intrachain interactions such as hydrogen bonds were destroyed (Figure S8), **PUE** chains partially extended, and the conjugation also extended to a certain extent. More importantly, a large number of noncovalent interactions and short contacts occur between adjacent **PUE** chains, especially in the alkoxy end regions (Figure S12). These interchain TSIs and short contacts are conducive to the formation and stability of large clusters.

### 2.5 Application

**Figure 5:**
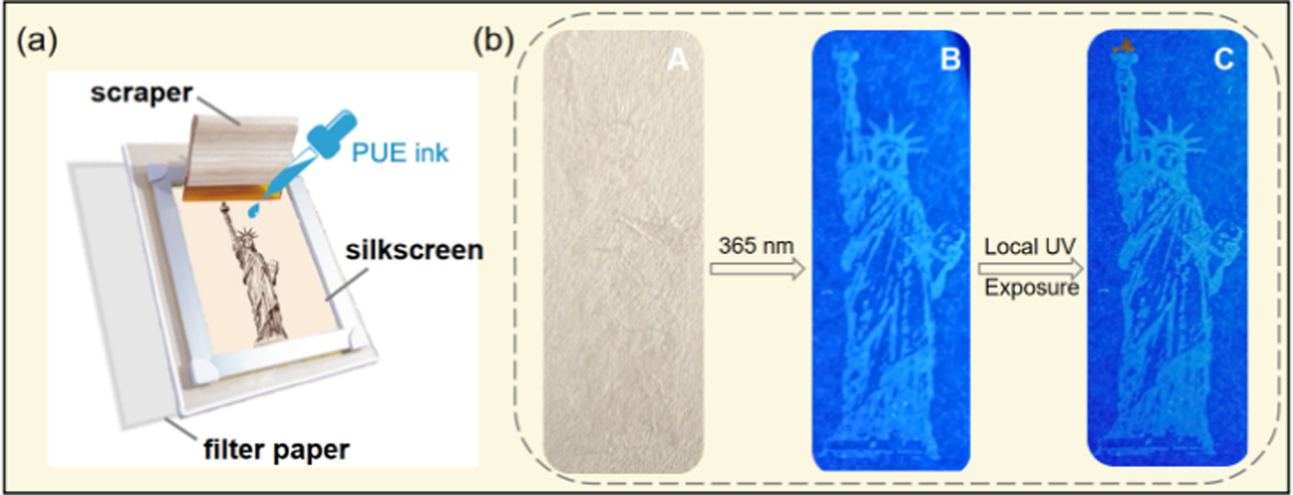
Schematic illustration of the application process for screen printing. (a) Schematic diagram of a screen-printing device. (b) Silkscreen image of the Statue of Liberty: (A) under daylight; (B) under 365 nm UV lamp; and (C) after local UV exposure for 10 min.

Thanks to the excellent solution processability, **PUE** can be used as a screen-printing ink. Based on the thermal/photochromic properties, **PUE** can be applied for encryption. An image of the Statue of Liberty printed on a filter paper through a screen-printing mold using **PUE-gel** as the ink is shown in Figure 5a. Figure 5b shows that under daylight, the pattern is barely visible on the filter paper. However, the fluorescent blue pattern of the Statue can be observed under a 365 UV lamp. When localized ultraviolet radiation is applied for 10 min, the torch in Lady Liberty’s hand lights up (Figure 5b(C))! This proof-of-concept model has the advantages of antireplication and high security, providing a new design for intelligent anticounterfeiting and information storage materials.

## 3 Conclusions

In summary, we have reported a novel multicolor aggregation-induced luminescence polyurethane derivative **PUE** driven by heat/light, successfully achieving noncontact multicolor fluorescence regulation of a monomolecular material. The luminescence behavior and the thermochromic/photochromic characteristics were systematically studied by photophysical experiments, FT-IR spectroscopy, WAXD experiments, and theoretical calculations. The flexible structure of **PUE** chains allows the regulation of multiple noncovalent intramolecular/intermolecular interactions of **PUE**. Under the stimulation of heat or UV light, hydrogen bonds in PUE chains break, curved molecular chains become extended, and conjugation can be increased. In addition, driven by a large number of noncovalent interactions, **PUE** chains further aggregate to form luminous clusters with varying degrees of aggregation. A large number of electronic interactions lead to exciton orbital overlap and energy-level splitting. Clusters with different degrees of aggregation bring different gaps and finally contribute to the multicolor emission behavior of **PUE**. The multicolor emission of the gel with heat- and light-induced aggregation is versatile for further developments in multifunctional stimulus-responsive macromolecules. Due to the excellent adhesive and high photochromic stability, **PUE-gel** can form fluorescent staining traces that are retained for a period of time. Therefore, **PUE-gel** has a broad application prospect in luminescent paints for security applications, signage, commodity items, automobiles, ships, or aircraft.

## 4 Experimental Section

### 4.1 Synthesis

**PUE** was prepared according to the following procedure. A mixture of estradiol (0.7136 g, 2.62 mmol), poly(ethylene glycol) monomethyl ether (*M*_w_ = 200 g mol^–1^; 0.396 g, 1.98 mmol), anhydrous THF (8 mL), trimethylhexa-1,6-diyl diisocyanate (0.7590 g, 3.61 mmol), and 1,4-diazabicyclooctane triethylenediamine (DABCO) (0.012 g, 0.06 mmol) was stirred in N_2_ atmosphere at 68 °C for 8 h until the clear solution became viscous, indicating that oligo/polymerization had occurred. After cooling to room temperature, the mixture was added to excess *tert*-butyl methyl ether drop by drop for reverse precipitation to give a product, which was then dried under a vacuum at room temperature for 24 h to obtain polyurethane **PUE** (1.21 g, 65% yield). ^1^H NMR (400 MHz, DMSO-*d*_6_, δ [ppm]): 7.76 (s, 2H), 7.64 (s, 2H), 7.59 (s, 1H), 6.65–7.35 (broad, 2H), 4.03 (s, 4H), 3.39–3.59 (broad, PEG protons), 3.23 (s, 6H; PEG terminal –OCH_3_ protons), 1.96–2.36 (broad, 2H), 1.05–2.35 (broad, 30H), 0.6–1.05 (broad, 9H). FT-IR: 3341 cm^–1^ (N–H), 2871 cm^–1^ and 2939 cm^–1^ (−CH_2_– asymmetric and symmetric stretch), 1718 (C=O), 1140 cm^–1^ (C–O–C stretch PEG). *M_n_* = 3696 g mol^–1^, *M*_w_ = 4866 g mol^–1^, PDI = 1.32.

### 4.2 Photophysical Characterization

The UV–vis absorption spectra were recorded on a Shimadzu UV-3100 spectrophotometer. The fluorescence spectra were recorded on a Hitachi model F-4700 spectrometer. The fluorescence lifetimes (τ) and fluorescence quantum yields (Φ_p_) were recorded using an Edinburgh Instruments FLS-1000 spectrometer. The luminescence photos were taken by an iPhone 14 pro under the irradiation of a hand-held UV lamp at room temperature.

### 4.3 Experimental Sample Preparation

**PUE-sol** was prepared by dissolving **PUE-powder** (30 mg) in dimethyl sulfoxide (DMSO) solution (2 mL). **PUE-gel** was prepared by dissolving 5 wt % **PUE-powder** in DMSO under heating, and after cooling to room temperature, **PUE-gel** was obtained.

### 4.4 Theoretical Calculations

The density functional theory (DFT) calculations were performed by using the Gaussian program. Two repeating molecular units with H as the chain ends were selected as the computational model. The structures of polyurethane with different conformations (helical and folded) were optimized at the B3LYP/6-31G* level with Gaussian 09 (D.01). The short contacts and hydrogen-bonding interactions were obtained at the B3LYP/6-311G** level with Gaussian 16 Revision B01.

## Supporting Information Available

Additional structural characterization; copies of NMR spectra; FT-IR spectra; additional photophysical data; scanning electron microscopy (SEM); and wide-angle X-ray diffraction (WAXD) data (PDF)
