# Supplementary material for: Multicolor Luminescence of a Polyurethane Derivative Driven by Heat/Light-Induced Aggregation
Source: Macromolecules. 2023 Sep 29;56(19):7721–8. doi: 10.1021/acs.macromol.3c01345 (PMC10569097; doi:10.1021/acs.macromol.3c01345)
Supplement: Supplementary file 1 — ma3c01345_si_001.pdf [file ma3c01345_si_001.pdf]

Supporting Information

Multicolor luminescence of a polyurethane derivative driven by heat/light - induced aggregation

Nan Jiang,† Ke-Xin Li,† Wei Xie,† Shu-Ran Zhang,† Xin Li,† Yue Hu,† Yan-Hong Xu,*,† Xing-Man Liu,*,# and Martin R. Bryce*,§

† Key Laboratory of Preparation and Applications of Environmental Friendly Materials, Key Laboratory of Functional Materials Physics and Chemistry of the Ministry of Education (Jilin Normal University), Changchun, 130103, China.

School of Chemistry and Chemical Engineering, Ningxia University, Yinchuan 750021, China

§ Department of Chemistry, Durham University, Durham DH1 3LE, UK

Contents:

1. Experimental - general information

2. Synthetic scheme

3. Photophysical properties and other structural characterization

4. Theoretical calculations

1. Experimental - general information

Materials obtained from commercial suppliers were used without further purification unless otherwise stated. All glassware, syringes, magnetic stirring bars, and needles were thoroughly dried in a convection oven. ^{1}H NMR spectra were recorded on a Varian 500 MHz spectrometer. The ^{1}H NMR spectra were referenced internally to the residual proton resonance in DMSO-d_6 (δ 2.5 ppm). The molecular weights of the polyurethane were determined by gel permeation chromatography (GPC) on a Waters 410 instrument with monodispersed polystyrene as the reference and THF as the eluent at 35 °C. Scanning electron microscope (SEM) images were obtained using a JEOL model JSM-6700 instrument operating at an accelerating voltage of 5.0 kV. The samples were prepared by placing microdrops of the solution on a holey carbon copper grid. Steady-state photoluminescence spectra were measured using a Hitachi F-4700 spectrometer. The fluorescence lifetime and photoluminescence quantum efficiency were obtained on an Edinburgh Instruments FLS-1000 instrument.

2. Synthetic scheme

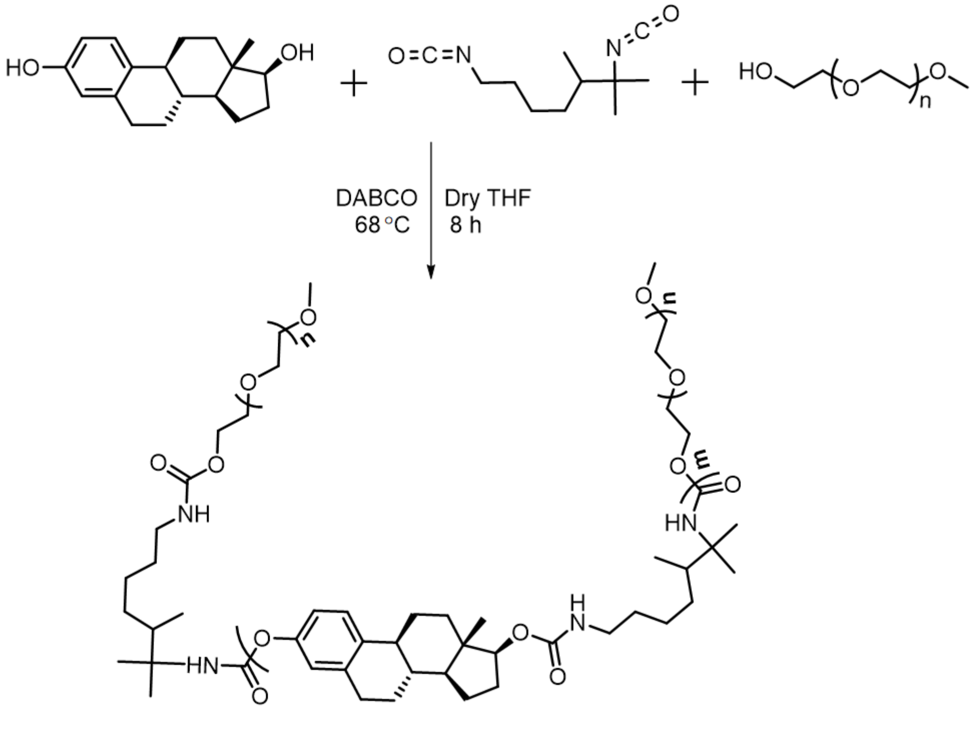

Figure S1. Synthetic route for **PUE**.

3. Photophysical properties

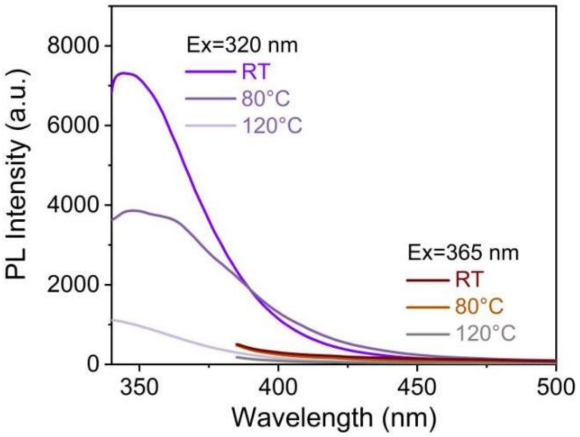

Figure S2. PL spectra of estradiol monomer at different excitation wavelengths.

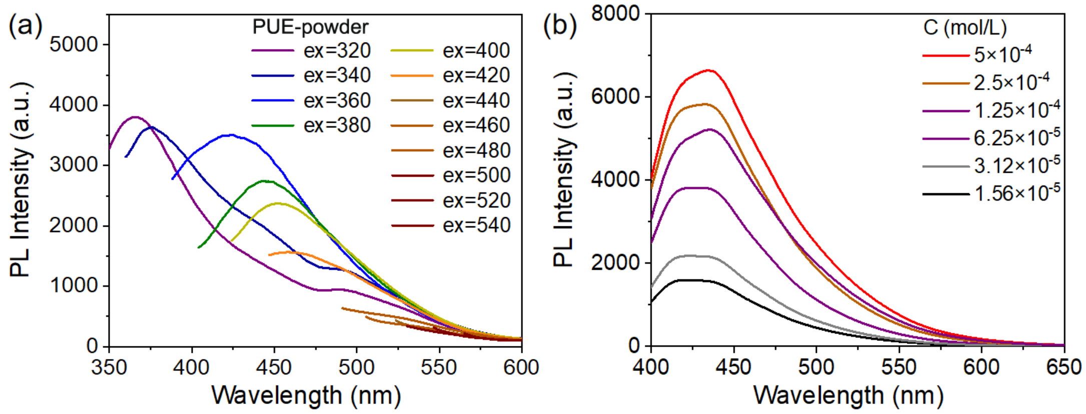

Figure S3. (a) PL spectra of **PUE-powder** at varying λ_{ex}. (b) PL spectra of **PUE**/trichloromethane with different concentrations.

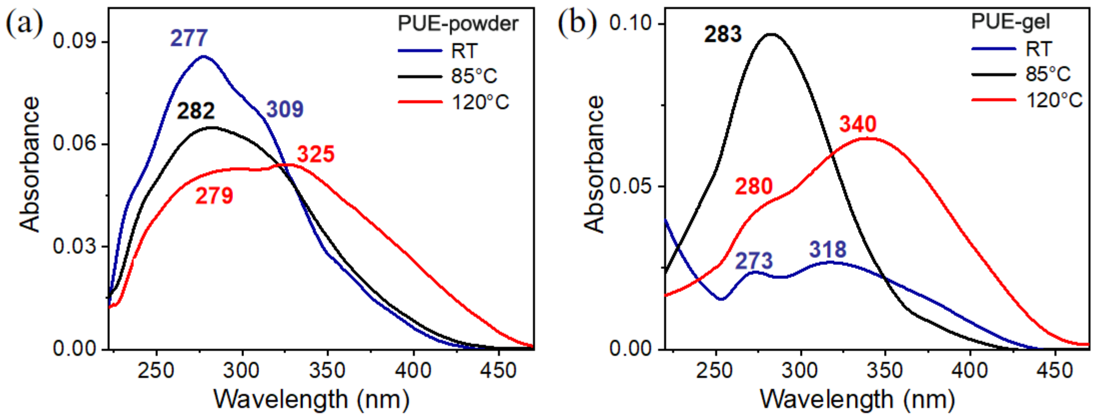

Figure S4. UV-vis absorption spectra (a) **PUE-powder** and (b) **PUE-gel** at different temperatures.

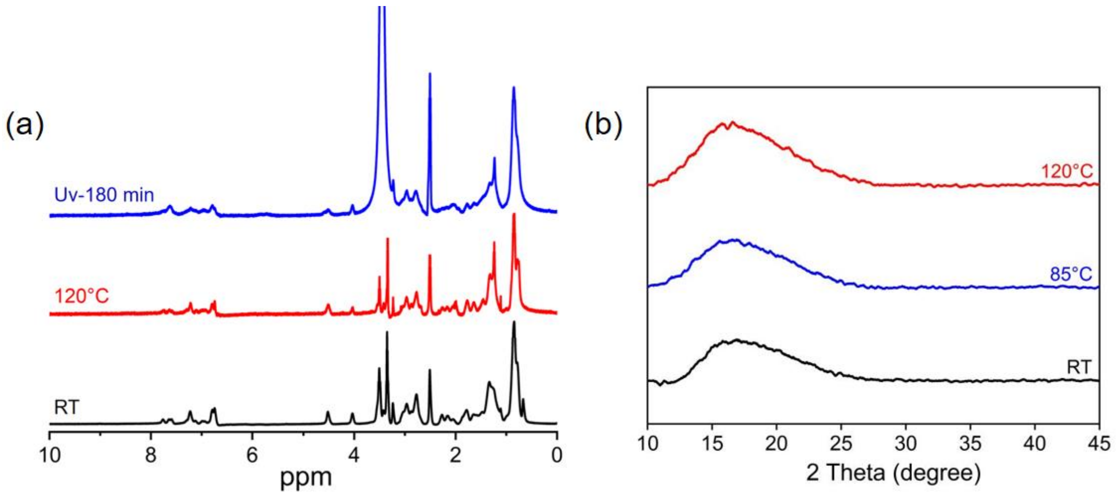

Figure S5. (a) ^{1}H NMR spectrum of **PUE** before and after heat/light treatment; (b) WAXD spectra of **PUE-powder** at room temperature and after heating to 85°C and 120°C.

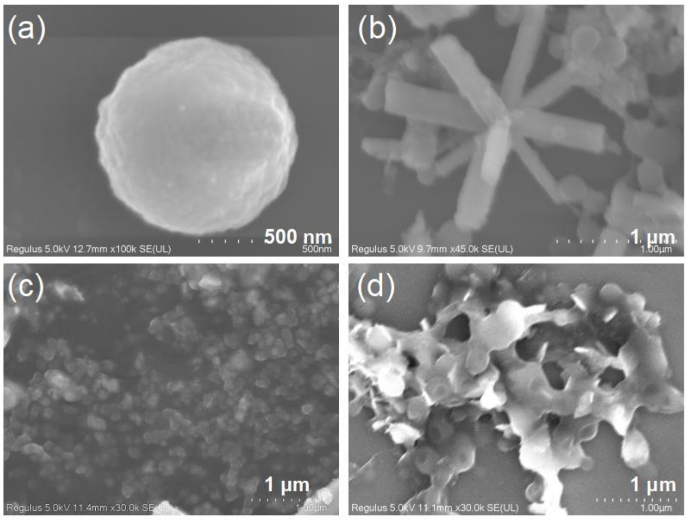

Figure S6. SEM images of (a) **PUE-gel** after heating at 85°C; (b) **PUE-gel** after heating at 120°C; SEM images of **PUE-powder** after ultraviolet irradiation (c) for 1 h and (d) for 2 h.

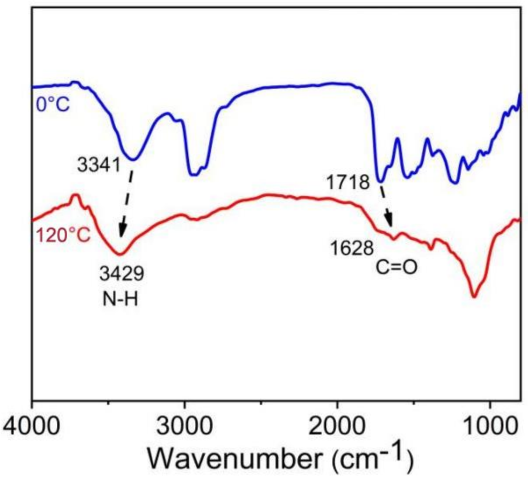

Figure S7. FT-IR spectra of **PUE-powder** before and after heating.

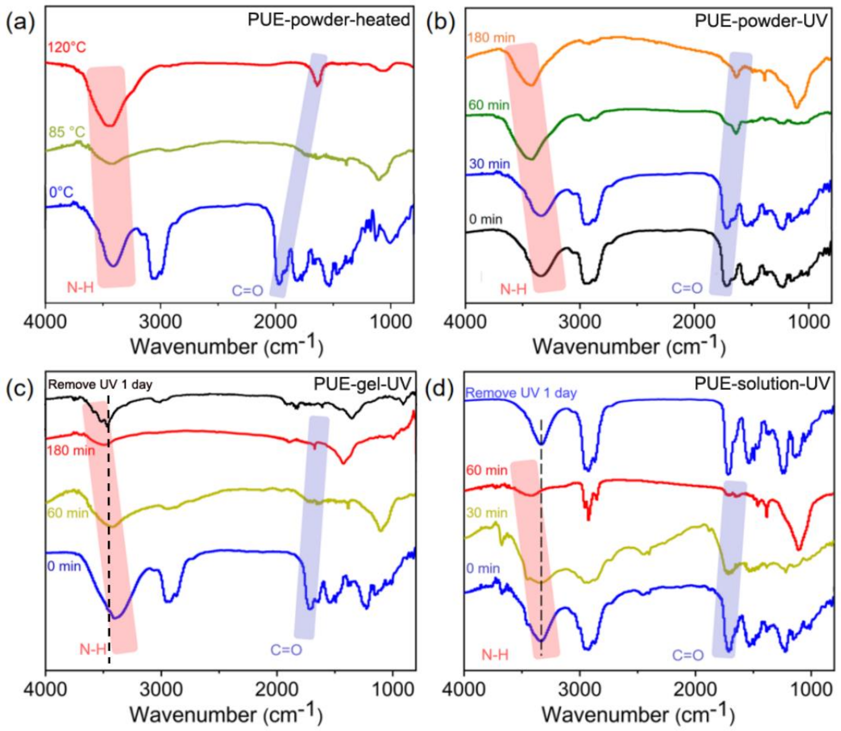

Figure S8. (a) FT-IR spectra of **PUE-powder** before and after heating. (b) FT-IR spectra of **PUE-powder** before and after ultraviolet irradiation; (c) FT-IR spectra of **PUE-gel** before and after ultraviolet irradiation; (d) FT-IR spectra of **PUE-sol** before and after ultraviolet irradiation.

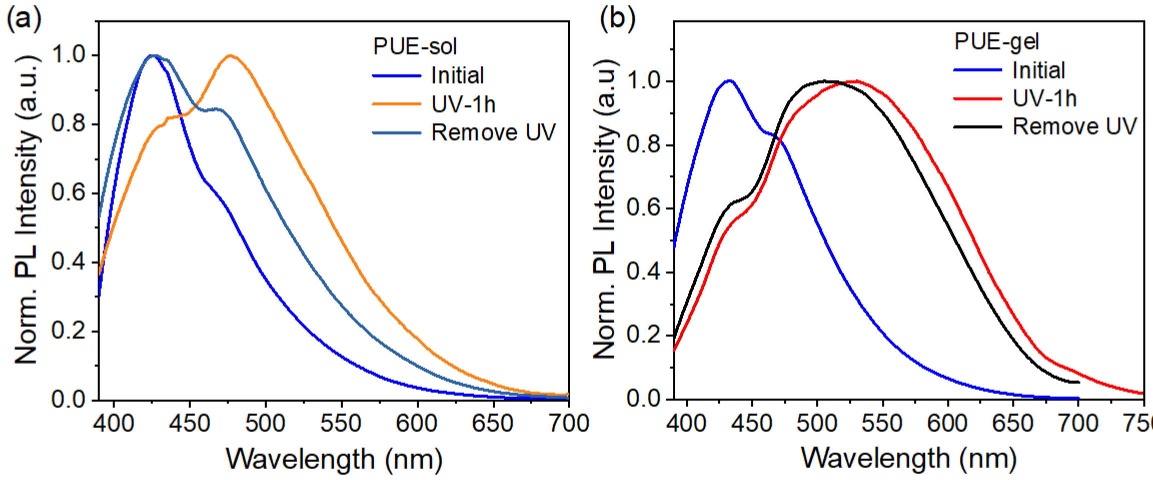

Figure S9. (a) PL spectra of **PUE-sol** before (initial) / after UV irradiation for 1 h and after storage in daylight at room temperature for 1 day. (b) PL spectra of **PUE-gel** before (initial) / after UV irradiation for 1 h and after storage in daylight at room temperature for 1 day.

Table S1. The fluorescence quantum yield (QY) and excited state lifetime (LT) of **PUE** in different states (λ_{ex}=365 nm).

PUE-sol:

	**LT**	**QY**
**Initial**	5.21 ns (λ_{em}=430 nm)	0.3%
**UV-1 h**	115 ns (λ_{em}=474 nm)	0.4%
**Remove UV 1 day**	4.01 ns (λ_{em}=432 nm)	0.3%

PUE-powder:

	**LT**	**QY**
**RT**	17.09 ns (λ_{em}=423 nm)	4.1%
**80°C**	4.60 ns (λ_{em}=464 nm)	4.0%
**120°C**	4.07 ns (λ_{em}=520 nm)	2.4%

PUE-gel:

	**LT**	**QY**
**RT**	1.52 ns (λ_{em}=436 nm)	1.0%
**80°C**	2.75 ns (λ_{em}=465 nm)	8.0%
**120°C**	4.51 ns (λ_{em}=522 nm)	2.1%

4. Theoretical calculations

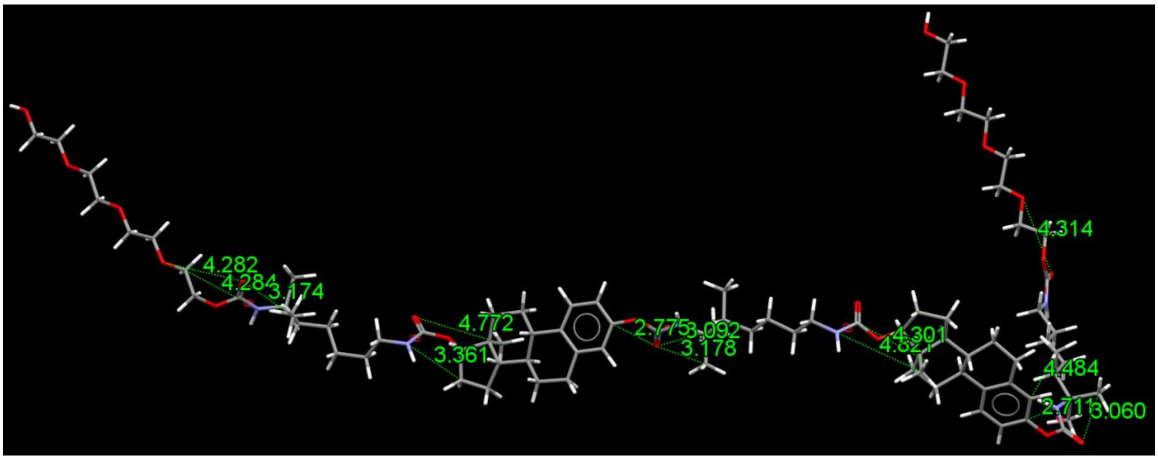

Figure S10. Theoretical calculations based on the single **PUE** chain model of weakly folded conformation obtained by G09.D01 at the B3LYP/6-31G(d) level.[S1]

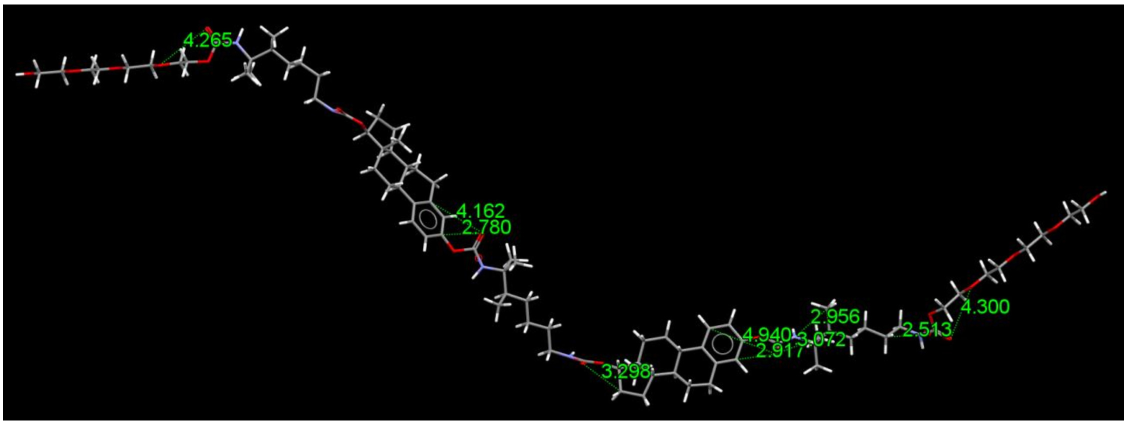

Figure S11. Theoretical calculations based on a single **PUE** chain model of non-folded conformation obtained by G09.D01 at the B3LYP/6-31G(d) level.[S1]

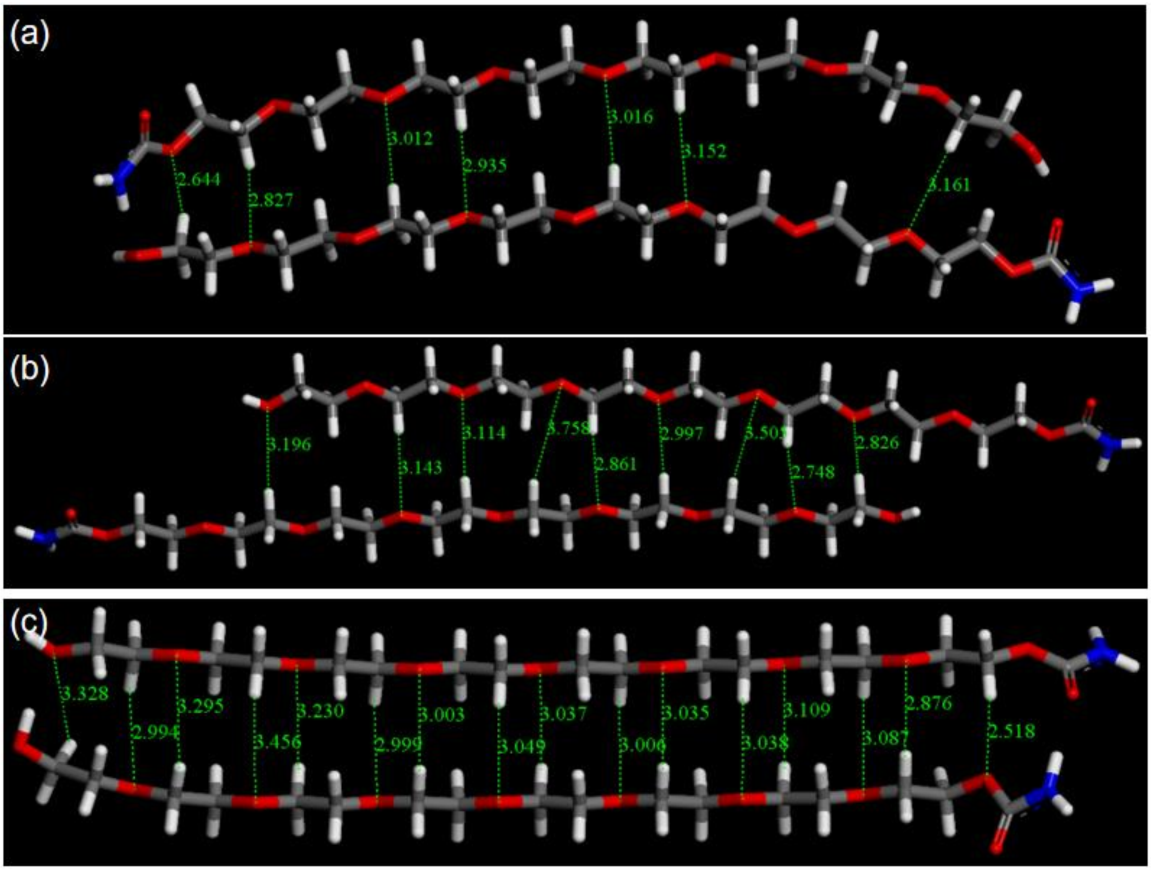

Figure S12. Diagram of short contacts and hydrogen-bonding interactions of alkoxy end parts in different conformations at the B3LYP/6-311G(d,p) level obtained by G16. B01. (a) Trans; (b) Dislocations; (c) Cis.[S2]

References

S1. Frisch, M. J.; Trucks, G. W.; Schlegel, H. B.; Scuseria, G. E.; Robb, M. A.; Cheeseman, J. R.; Scalmani, G.; Barone, V.; Mennucci, B.; Petersson, G. A. et al. Gaussian 09 (Revision D.01), Gaussian, Inc., Wallingford, Connecticut, 2009.

S2. Frisch, M. J.; Trucks, G. W.; Schlegel, H. B.; Scuseria, G. E.; Robb, M. A.; Cheeseman, J. R.; Scalmani, G.; Barone, V.; Petersson, G. A.; Nakatsuji, H. et al. Gaussian 16 (Revision B.01); Gaussian, Inc., Wallingford, Connecticut, 2016.
